# Protective Effect of Nanobodies Targeting Sip Protein Against *Streptococcus agalactiae* Infection in Tilapia (*Oreochromis niloticus*)

**DOI:** 10.3390/ani15213207

**Published:** 2025-11-04

**Authors:** Zhishen Wang, Huiling Wu, Weihao He, Shunqiang Wei, Xuemin Wei, Chaoshuai Wei, Yinghui Wang, Aiguo Huang

**Affiliations:** 1 Guangxi Laboratory on the Study of Coral Reefs in the South China Sea, Coral Reef Research Center of China, School of Marine Sciences, Guangxi University, Nanning 530004, China; 2227301031@st.gxu.edu.cn (Z.W.); scauheweihao@163.com (W.H.); 18577889437@163.com (S.W.); 2327301032@st.gxu.edu.cn (X.W.); wcs@gxu.edu.cn (C.W.); 2 Institute of Green and Low Carbon Technology, Guangxi Institute of Industrial Technology, Nanning 530000, China; whl415899@126.com

**Keywords:** nanobodies, *Streptococcus agalactiae*, surface immunogenic protein, *Oreochromis niloticus*, antibacterial activity

## Abstract

**Simple Summary:**

Antibiotics remain the primary method for controlling *Streptococcus agalactiae* (GBS) in aquaculture environments. However, while antibiotics can eliminate GBS, they may also lead to the release of virulence factors and the emergence of drug-resistant strains. Additionally, GBS can breach the blood–brain barrier in fish and damage the nervous system, accelerating fish mortality. Therefore, this study aims to explore green, virulence-neutralizing, and blood–brain barrier-penetrating strategies for preventing GBS infection. Nanobodies (Nbs), currently the smallest genetically engineered antibodies, possess potential for rapid degradation, toxin neutralization, and barrier system penetration, making them a crucial source for new drug development. Notably, this study utilized phage display technology to screen and obtain a nanobody Nb30 from a camel-derived nanobody phage library, which can target surface immunogenic (Sip) protein and inhibit GBS infection in tilapia (*Oreochromis niloticus*) tissues, and improve survival rates in infected fish. In summary, Nb30 is a sustainable, effective neutralizing antibody with promising application potential.

**Abstract:**

*Streptococcus agalactiae* (GBS) has emerged as one of the most prevalent bacterial pathogens causing severe economic losses in tilapia aquaculture due to its highly contagious and lethal nature. Nanobodies (Nbs), characterized by their small molecular size, enhanced tissue penetration, high tolerance, and exceptional antigen-binding affinity, represent a promising green alternative to conventional antibiotics. In the present study, the objective was to explore the potential of specific Nbs in the treatment of tilapia GBS disease. We first screened specific Nbs targeting the surface immunogenic (Sip) protein of GBS from a naïve phage display library, and a novel nanobody Nb30 was obtained. Nb30 was expressed in *Escherichia coli* and purified using the Ni-NTA Agarose column. Indirect ELISA showed that Nb30 had a high affinity against Sip and GBS in vitro. Moreover, Nb30 significantly reduced GBS colonization in the liver, spleen, and brain of GBS-infected tilapia. The survival rate in the control groups was 53%, whereas it was increased to 86% after treatment with 100 mg/kg Nb30. Transcriptome profiling revealed that Nb30 could modulate critical biological processes, including antioxidant defense, immune regulation, amino acid/protein synthesis, and energy metabolism in the liver tissues of GBS-infection tilapia. Notably, the expression levels of antioxidant enzymes (*cat* and *gpx*) were significantly up-regulated, and the TLR/MyD88/NF-κB pathway-related genes (*tlr5*, *myd88*, *irak4*, *traf6*, *Rela*, and *NF-κB2*) were significantly down-regulated after treatment with Nb30. Collectively, these findings establish a novel therapeutic strategy for controlling GBS infection in tilapia and provide evidence supporting the application of nanobodies as sustainable alternatives to antibiotics in aquaculture disease management.

## 1. Introduction

Tilapia is one of the most important aquaculture species recommended by the Food and Agriculture Organization of the United Nations (FAO) to the world [1]. However, in recent years, with the expansion of aquaculture scale and the increase in aquaculture density, the prevalence of bacterial infections, primarily caused by *Streptococcus agalactiae,* has posed a significant threat in major tilapia farming regions globally [2,3]. *S*. *agalactiae*, also referred to as Group B *Streptococcus* (GBS), can be categorized into 10 serotypes, Ia, Ib, and II-IX depending on the capsule polysaccharide [4]. The main signs of GBS infection in tilapia are septicemia and meningitis, resulting in melanosis, exophthalmia and erratic swimming [5,6]. In the past, broad-spectrum antibiotics have been used commercially for the treatment of GBS infection in tilapia, but the misuse of antibiotics has resulted in the widespread occurrence of bacterial resistance, which affects their therapeutic efficacy and contributes to environmental pollution [7,8]. Therefore, there is an urgent need for the development of a novel environmentally friendly alternative to antibiotics for GBS infection.

In the 1990s, Hamers and his colleagues identified a naturally occurring monoclonal antibody in camelid serum that lacked the light chain. The cloning of the variable region of the heavy chain antibody resulted in the generation of a single-domain antibody, also referred to as a nanobody (Nb), which was constituted exclusively of heavy chains [9]. Nbs are the smallest antigen-binding fragments known to exist naturally, and they have the advantages of small molecular weight, high tissue penetration, high tolerance, high affinity, and ease of production. The excellent properties of Nbs have promoted their applications in the fields of neutralizing viruses and pathogenic bacteria [10,11,12]. For example, Nbs could target the virulence factor FliC protein against *Salmonella enteritidis* infection in chicken [13]. In addition, Nbs could effectively interact with lipopolysaccharides and neutralize *Vibrio cholerae* infection in vitro and in vivo [14]. These studies suggest that the development of Nbs against virulence proteins or surface components of pathogenic bacteria can prevent and treat pathogen infection. Therefore, Nbs may be a new alternative to antibiotic treatment.

Adhesins play a key role in the process of bacterial invasion into the host by interacting with corresponding receptors on the host cell to achieve bacterial attachment [15]. Studies have shown the presence of a variety of adhesins on the surface of GBS, including fibrinogen-binding proteins (Fbs), laminin-binding proteins (Lmb), C5a peptidase (ScpB), and surface immunogenic protein (Sip) [16]. Sip, as a major adhesin of GBS, exhibits high homology across different serotypes of GBS and is involved in bacterial adhesion and host tissue colonization. It has been noted that by knocking out the gene encoding Sip, the virulence of the obtained GBS strains was significantly reduced [17]. Thus, the Sip has become a main target for therapeutic development. Sip protein has been shown to a strong immunogenicity in a variety of animal models for immune experiments. An animal model demonstrated that specific antibodies against Sip could cross the placenta to confer protection to newborns against diseases caused by GBS [18].

In this study, we aimed to screen Sip-targeting nanobodies (Nbs) from a naïve phage-displayed nanobody library through three rounds of panning-elution-amplification cycles. The selected Nbs were subsequently expressed and purified. In vitro experiments were used to demonstrate the binding capability of purified Nbs to Sip and GBS. Furthermore, the GBS-infected tilapia (*O*. *niloticus*) model was utilized to evaluate the in vivo antibacterial activity of Nbs. The mechanisms underlying their anti-GBS effects and protective roles in tilapia were systematically investigated. This study would provide a novel strategy for disease prevention and control of GBS infections in aquaculture.

## 2. Materials and Methods

### 2.1. Fish and Bacteria

Healthy tilapia (9 ± 0.5 g) were purchased from a tilapia farm in Nanning, Guangxi, China. Throughout the study, these fish were kept in 100 L aeration tanks and fed a commercial diet twice a day. The water was partially changed daily and the temperature was maintained at 30 ± 1.0 °C. The animal use protocol was approved by the Animal Care and Welfare Committee of Guangxi University (GXU-2023-0134). The Ia-type GBS isolated from the tilapia fish farm in Nanning, Guangxi was generously donated by Dr. Song Zhu from Southwest University. The *Escherichia coli* SS320 strain and the helper phage M13K07 were obtained from Kangti Life (Shenzhen, China).

### 2.2. Biopanning of Sip-Specific Nbs from Phage Display Libraries

The purified Sip protein of GBS was previously prepared and stored in our laboratory according to methods described by previous researchers [19]. The selection process of nanobodies, as previously described [20], can be summarized as follows: the immunotubes are coated with purified Sip protein (50 μg per tube) at 4 °C overnight, and a negative blank control was established to account for nonspecific binding. Immunization tubes were washed 3 times with PBS, followed by the addition of 2 mL of blocking solution (5% skimmed milk powder) and spin blocking for 2 h. Naïve phage-displayed Nb libraries (1 × 10^12^ pfu/tube) were added to the immunization tubes and incubated at room temperature with spinning for 1 h. The tubes were washed 20 times by the addition of 2 mL of PBST (1× PBS, containing 0.05% Tween 20 (Solarbio, Beijing, China)). The remaining bound phage particles were eluted with 1 mL of 0.25 mg/mL Trypsin (Sangon Biotech, Shanghai, China) solution for 30 min and terminate the elution by adding 10 μL of 10% AEBSF (Sangon Biotech, Shanghai, China). *E. coli* SS320 was infected with the eluted phage with M13K07 helper phage and subsequently amplified overnight at 37 °C. The amplified phage was finally purified using PEG 4000/2.5 M NaCl (Solarbio, Beijing, China) precipitation for the next round of selection. The purified phages were identified using Phage ELISA as previously reported [20]. In brief, the recombinant Sip (approximately 400 ng/well) was coated on a 96-well plate at 4 °C. After washing three times with PBST, the plate was blocked with 1.5% skim milk at 37 °C for 1 h. The plate was then washed and the amplified phage was added to each well, followed by incubation at 37 °C for 2 h. The plate was washed six times with PBST, and then the HRP-conjugated anti-M13 mouse monoclonal antibody (Santa Cruz, Shanghai, China) was added, incubated at 37 °C for 1 h. Subsequently, 100 μL of TMB two-component color development solution (Solarbio, Beijing, China) was added, and the reaction was allowed to develop. The reaction was stopped by adding 50 μL of ELISA stop solution (Solarbio, Beijing, China), and the absorbance was measured at 450 nm using a microplate reader. The sequencing of clones that yield a positive reaction with phiS3/psiR3 primers (Table S1), and the grouping of Nbs based on differences in the CDR regions.

### 2.3. Expression and Purification of Nanobody Nb30

A pair of primers Nbs-F/Nbs-R (Table S1) was designed based on the gene sequence of Nb30, and PCR amplification was performed to amplify the Nb30 gene. The amplified product was cloned into the EcoRI and HindIII sites of the pET-28a (+) vector. The recombinant plasmid pET28a-Nb30 was then transformed into *Escherichia coli* BL21(DE3) (Sangon Biotech, Shanghai, China). Expression of the recombinant protein Nb30 was induced with 0.8 mM IPTG (Sangon Biotech, Shanghai, China) at 37 °C for 6 h. Cells were disrupted using a cell disruptor, and the supernatant and pellet fractions were analyzed by SDS-PAGE. The inclusion body protein was solubilized in 8 M urea and purified using a Ni-NTA affinity chromatography column (CWBIO, Taizhou, China). The refolding of the denatured protein was performed according to the manufacturer’s protocol. Briefly, the inclusion body protein was dialyzed against 8 M→6 M→4 M→2 M→0 M urea solutions at 4 °C for 12 h each step, with the final dialysis in PBS buffer. The refolded protein was concentrated using a centrifugal filter and the concentration was determined using a BCA protein assay kit (CWBIO, Taizhou, China).

### 2.4. In Vitro Affinity Validation

To detect the binding activity of purified Nb30 to Sip and GBS, an indirect ELISA was performed as described in a previous report [20]. Briefly, 96-well plates were immobilized with purified Sip protein (400 ng per well) or inactivated GBS (about 1 × 10^8^ CFU/mL). Then a series of purified recombinant protein Nb30 (0.05 to 10 mg/L) was added to the plates and incubated with Sip or GBS. Finally, an anti-His tag monoclonal antibody (Solarbio, Beijing, China) was used to detect the bound Nb30.

### 2.5. Anti-GBS Activity of Nb30 in Tilapia

The anti-GBS activity of Nbs was tested by analyzing the bacterial copy number in tilapia tissues and the survival rate of GBS-infected tilapia. Tilapia were randomly divided into three groups: control groups, low-dose treatment groups and high-dose treatment groups. The control groups were injected intraperitoneally with 100 μL of GBS solution (about 3 × 10^8^ CFU/mL), the low-dose groups were injected with 100 μL of mixtures containing GBS and 50 mg/kg of Nb, and the high-dose groups were injected with GBS and 100 mg/kg of Nb. Each group was performed in triplicate, and each replicate contained 15 fish. After 24 h of treatment, tilapia were euthanized and the liver, spleen, and brain tissues were sampled and stored at −80 °C for subsequent experiments. The same treatment was performed to evaluate the protective effects of Nb30 on GBS-infected tilapia.

### 2.6. Genomic DNA Extraction and Absolute Quantification of GBS Copy Numbers

Using a tissue grinder to perform physical grinding on the tilapia tissue, and total genomic DNA was extracted using the TIANamp Bacteria DNA Kit (Tiangen, Beijing, China) according to the manufacturer’s instructions. GBS genomic DNA was quantified by real-time qPCR (qPCR) as described in a previous study [20]. Briefly, the *cfb* gene encoding the CAMP factor was selected as the target gene and cloned into the pMD19-T vector (Takara, Beijing, China) by using cfb primers (Table S1). The copy number of the target amplicon was quantified by measuring the concentration using a NanoDrop spectrophotometer (Analytik Jena, Jena, Germany). The target amplicon was diluted into a 10-fold series and used as a template for qPCR. qPCR was performed in a Rapid Real-Time Fluorescence Quantitative PCR System (ABI 7500, Thermo Fisher, Waltham, MA, USA) using UltraSYBR mixtures (CWBIO, Taizhou, China). qPCR cycling conditions were 95 °C for 10 min, followed by 40 cycles of denaturation at 95 °C for 15 s, and then annealing at 60 °C for 1 min. To verify the amplification of individual products, melt curves were analyzed at the end of each PCR thermal profile from 65 °C to 95 °C in 5 s steps. The logarithm of the *cfb* gene copy number and the corresponding cycling threshold (Ct) value were regressed as a standard curve to determine the copy number of GBS.

### 2.7. RNA Extraction and Relative Quantification of Tilapia Gene Expression

Total RNA was extracted from the tilapia liver tissues using TRIzon reagent (CWBIO, Taizhou, China). The mass and concentration of extracted RNA were determined using 1.5% agarose gel electrophoresis and a NanoDrop spectrophotometer. The extracted RNA was then reverse transcribed into cDNA using HiFiScript cDNA synthesis kit (CWBIO, Taizhou, China). The expression levels of *tlr5*, *myd88*, *irak4*, *traf6*, *Rela*, *NF-κb2*, *cat*, *gpx* and *c-type lysozyme* were detected by qPCR. *β-actin* was chosen as the reference gene in this study. The primers for qPCR in this study are shown in Table S1. The fold changes of gene expression levels were performed by the 2^−ΔΔCt^ method.

### 2.8. Library Construction and Transcriptome Sequencing

From the control group and high-dose treatment groups, 9 fish were randomly selected from each group. The liver tissue of these fish was isolated, and the liver tissue of 3 fish was mixed to form one sample. All samples were immediately frozen in liquid nitrogen and stored at −80 °C ultra-low temperature refrigerator. After the collection was completed, the samples were sent to Shanghai Meiji Biomedical Technology Co., Ltd. (Shanghai, China). for sample preparation and library construction to ensure the accuracy and reliability of the data. Total RNA was extracted from the liver tissue of the Nile tilapia using Trizol reagent and the RNA Purification Kit. First-strand cDNA was synthesized using random primers and reverse transcriptase, followed by second-strand cDNA synthesis using DNA polymerase and RNase H. The purified cDNA was end-repaired and A-tailed, and specific adapters were ligated to construct the sequencing library. The library was amplified and screened by PCR to verify appropriate fragment size and concentration. After quality control, high-throughput sequencing was performed on the Illumina NovaSeq X Plus sequencer (San Diego, CA, USA) [21].

Raw data were processed using Fastp (Version 0.23.4) to obtain high-quality clean data. The filtered data were aligned to the reference genome (GenBank: GCF_001858045.2) using appropriate parameters. Differential expression genes (DEGs) were identified using DESeq2 (Version 1.42.0) with default settings and thresholds of *p*-adjust < 0.05 and |log_2_FC| > 1. GO and KEGG enrichment analysis of DEGs was performed on the Majorbio Cloud Platform (https://www.majorbio.com/tools, accessed on 9 January 2025).

### 2.9. Statistical Analysis

Statistical analyses were performed using SPSS 18.0 statistical software (SPSS Inc., Chicago, IL, USA). Data were expressed as mean ± standard deviation (SD). The significance determined by analyzing the data was using one-way analysis of variance (ANOVA) and post hoc Tukey test. Significant differences were defined as *p* < 0.05 (*), *p* < 0.01 (**).

## 3. Results

### 3.1. Biopanning and Identification of Sip-Specific Nbs

**Figure 1:**
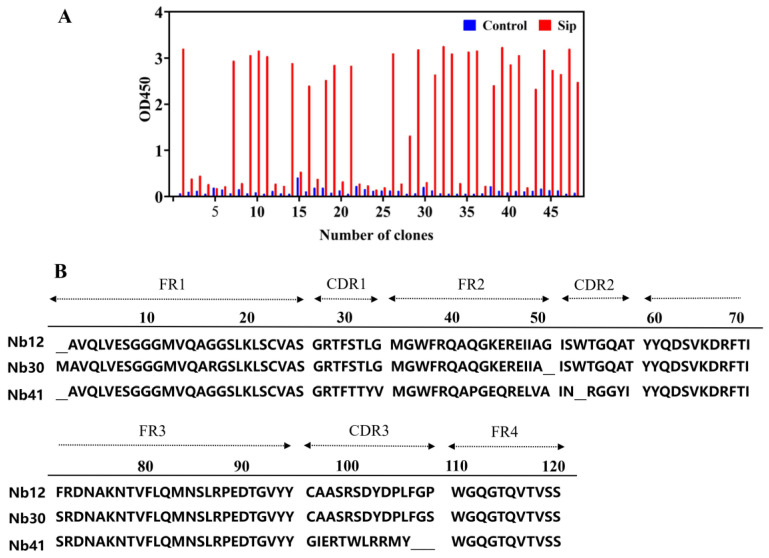
Identification of nanobodies (Nbs) targeting Sip. (**A**) Identification of specific Nbs by indirect phage ELISA; (**B**) Analysis of the amino acid sequence of positive Nbs.

The purification of the Sip protein was obtained using a Ni-NTA affinity column (Figure S1), and used as a target protein for biopanning of Nbs. After three rounds of panning-elution-amplification, 48 positive clone strains were identified using phage ELISA, and three Nb clones with different sequences, named Nb12, Nb30, and Nb41, were obtained after sequence alignment (Figure 1A). These Nb clones were similar in four framework regions, and differed significantly only in three CDR regions (Figure 1B). In the study, Nb30 was selected for further investigation based on its in vitro affinity activity.

### 3.2. Prokaryotic Expression, Purification and In Vitro Affinity Test of Nb30

**Figure 2:**
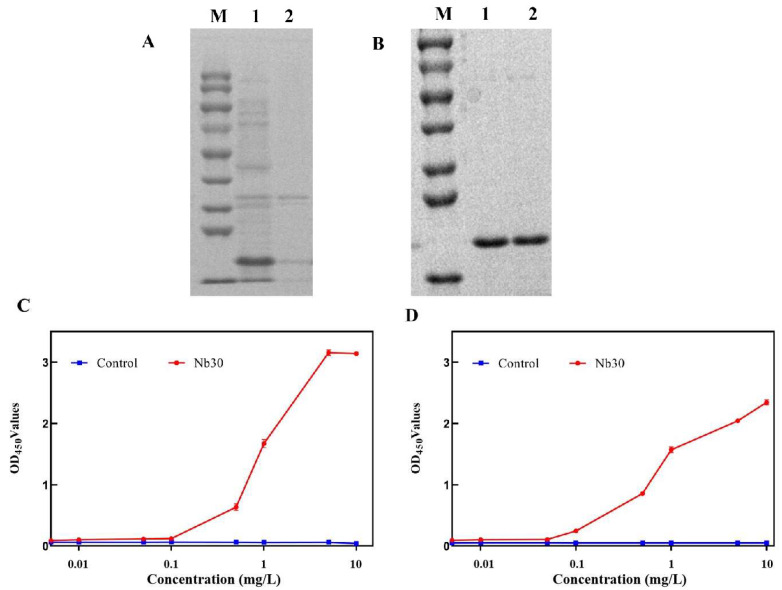
Expression and purification of nanobody Nb30. (**A**) SDS-PAGE analysis of the expression of Nb30. M: protein marker, lane 1: precipitation of Nb30 expression, lane 2: supernatant of Nb30 expression; (**B**) SDS-PAGE analysis of the purified Nb30; (**C**,**D**) Detection of the binding ability of Nb30 to (**C**) Sip and (**D**) GBS by indirect ELISA.

The results of SDS-PAGE showed that Nb30 was mainly expressed as inclusion bodies in BL21 cells, and its molecular weight was about 20 kDa (Figure 2A). Recombinant protein Nb30 was further purified using a Ni-NTA affinity column (Figure 2B). Subsequently, the binding activity of Nb30 to Sip or GBS was detected using ELISA. The results indicate that the in vitro-expressed Nb30 maintains binding activity with Sip and GBS, and its binding capacity increases with increasing concentrations of Nb30, exhibiting a clear dose-dependent effect (Figure 2C,D).

### 3.3. Nb30 Improves Survival Rate and Inhibits GBS Infection in Tilapia

**Figure 3:**
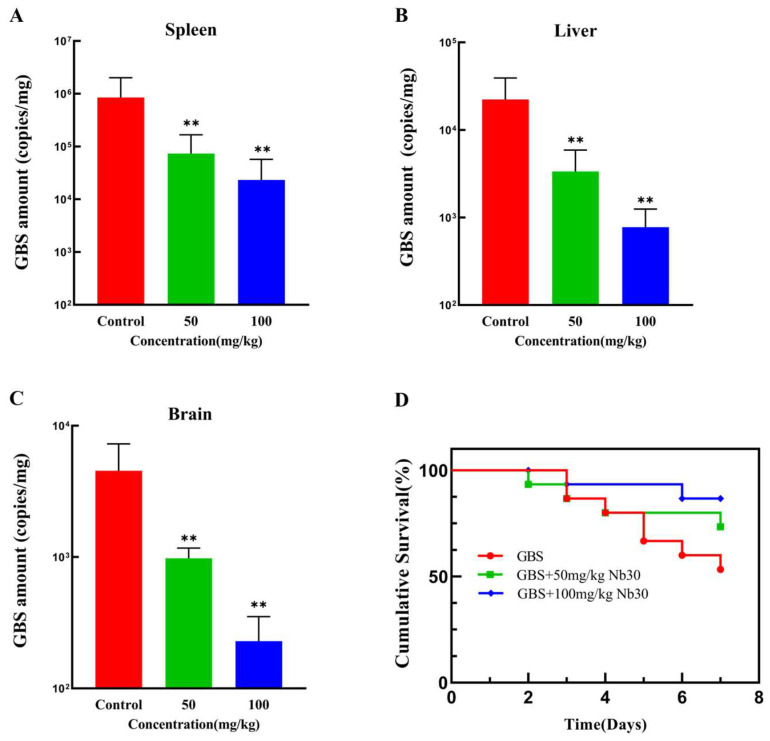
Antibacterial activity of Nb30 against GBS in tilapia. Copy numbers of GBS in (**A**) spleen, (**B**) liver, and (**C**) brain tissues of tilapia after 24 hpi; (**D**) Survival curves of GBS-infected tilapia treated with different concentrations of Nb30. Mortality in each group was recorded continuously for 7 days. ** *p* < 0.01, compared to control.

As shown in Figure 3A–C, Nb30 significantly inhibited GBS infection in different tissues of tilapia within 24 h at concentrations of 50 and 100 mg/kg. Compared with the control groups, the GBS copy numbers in spleen, liver and brain tissues of GBS-infected tilapia, respectively, decreased by 29.43, 7.62 and 5.84-fold after treatment with 50 mg/kg Nb30, and decreased by 45.84, 25.78 and 11.52-fold after treatment with 100 mg/kg Nb30. To investigate the protective effect of Nb30 on GBS-infected tilapia, a 7-day cumulative survival rate experiment was conducted. As shown in Figure 3D, there was a difference in the survival rate of GBS-infected tilapia between the control groups and treatment groups. The survival rate in the control groups was 53%, whereas the survival rate was increased to 73% (*p* > 0.05) and 86% (*p* > 0.05) after treatment with 50 mg/kg and 100 mg/kg Nb30.

### 3.4. Transcriptome Changes in the Liver of GBS-Infected Tilapia Under Nb30 Treatment

**Figure 4:**
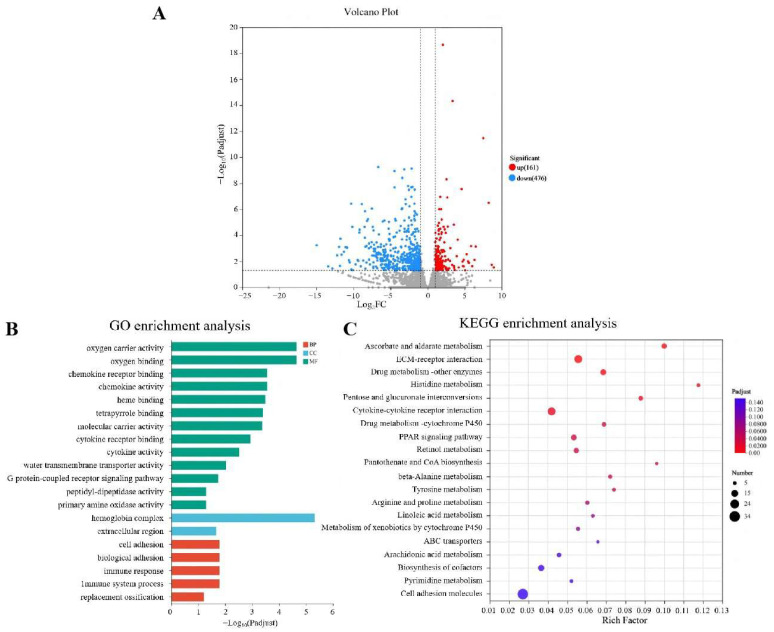
Integration diagram of multidimensional transcriptome analysis of tilapia liver tissue after treatment with Nb30 for 24 h. (**A**) Volcano plot of the comparison between the control group and high-dose treatment groups; (**B**) GO enrichment analysis of the differentially expressed genes (DEGs) in comparison of the control group and high-dose treatment groups; (**C**) KEGG enrichment analysis of the DEGs in comparison of the control group and high-dose treatment groups.

To further investigate the protective mechanism of Nb30 against GBS in tilapia, transcriptomic sequencing was performed between the control groups and high-dose groups (Figure 4). As shown in Figure 4A, a total of 637 significantly differential genes (DEGs) were identified, of which 161 genes were up-regulated and 476 genes were down-regulated. To predict the relevant biological functions and corresponding pathways of these important DEGs, GO and KEGG enrichment analyses were performed. GO functional enrichment analysis classified DEGs into three functional groups, and the top 20 DEGs that were significantly enriched in BP (Biological Processes), CC (Cellular components), and MF (Molecular Function) (Figure 4B). In the context of BP, DEGs were found to be enriched in “oxygen carrier activity”, “chemokine receptor”, “chemokine activity”, “molecular carrier activity”, “G protein-coupled receptor binding” and “heme binding”. About CC, DEGs were predominantly found to belong to the “hemoglobin complex” and “extracellular region”. In the MF, DEGs were mainly enriched in “immune system processes”, “immune response” and “cell adhesion”. KEGG analysis showed that the top 20 KEGG pathways were significantly enriched in “Ascorbate and aldarate metabolism”, “Pentose and glucuronate interconversions”, “Histidine metabolism”, “Retinol metabolism”, “beta-Alanine metabolism”, “Cytokine-cytokine receptor interaction”, “Drug metabolism-cytochrome P450”, “Metabolism of xenobiotics by cytochrome P450” and “PPAR signaling pathway”, the majority of which are associated with immune-related functions (Figure 4C).

### 3.5. Analysis of the Gene Expression of TLR/NF-κB Pathway, Antioxidant and Immune Levels in Tilapia

**Figure 5:**
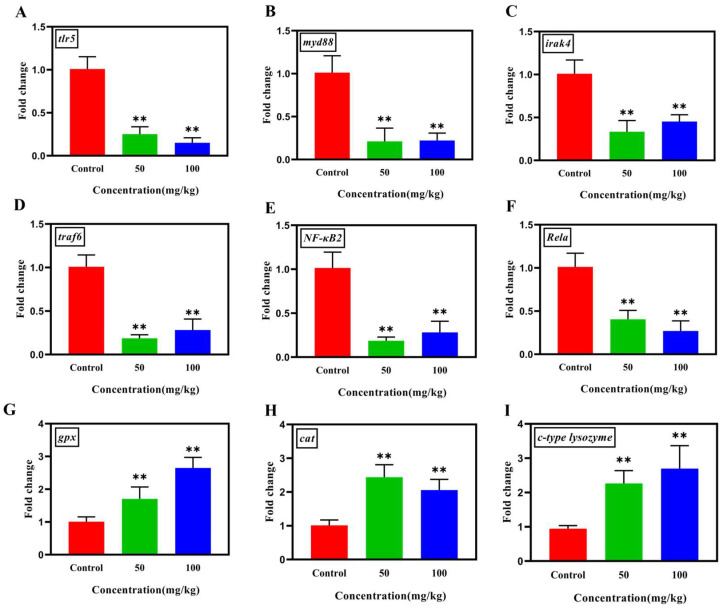
The effect of Nb30 on the expressions of TLR/MyD88/NF-κB inflammatory pathway, antioxidant and immune genes (**A**) *tlr5*, (**B**) *myd88*, (**C**) *irak4*, (**D**) *traf6*, (**E**) *Nf-kB2*, (**F**) *Rela*, (**G**) *gpx*, (**H**) *cat*, and (**I**) *c-type lysozyme* in the liver tissues of GBS-infected tilapia after 24 hpi. ** *p* < 0.01, compared to control.

Differences in the gene expressions involved in the TLR/MyD88/NF-κB inflammatory pathway were investigated in the liver tissues of tilapia in this study. As shown in Figure 5A–F, the transcriptional levels of genes *tlr5*, *myd88*, *irak4*, *traf6*, *Rela,* and *NF-kB2* within TLR/NF-κB pathway in high-dose groups were significantly down-regulated (*p* < 0.01) compared with those in the control groups. Moreover, the antioxidant genes *cat* and *gpx*, and the immune gene *c-type lysozyme* were significantly up-regulated (*p* < 0.01) after Nb30 treatment (Figure 5G–I).

## 4. Discussion

GBS, a serious pathogen, poses a major threat to the tilapia aquaculture industry. In the context of diminishing effectiveness of antibiotic treatments, the development of new prevention and control strategies is particularly urgent. Vaccination has received much attention as an emerging effective tool [22,23]. However, due to the diversity of GBS serotypes, designing and preparing vaccines that offer broad protection has become a major challenge in vaccine development. Sip protein, one of the key adhesion proteins for the invasion of host cells by GBS, is highly conserved, making the Sip proteins a popular target for the development of vaccines or drugs against GBS. Currently, delivery of purified Sip proteins to tilapia in a variety of ways to stimulate the production of specific antibodies is an effective method against GBS infestation [24,25]. However, the method has the limitation that inoculation is time-consuming. Nanobodies have great advantages in the diagnosis and treatment of bacterial and viral diseases because their elongated CDR3 loop provides sufficient antigen-binding sites and can extend to hidden epitopes of antigens [26], making the development of nanobodies targeting Sip proteins a promising new antibacterial strategy.

Phage display technology is currently the mainstream technique for screening nanobodies (Nbs) against target antigens, which allows Nbs to be displayed on the phage surface and maintain their spatial structure and bioactivity, and this approach greatly improves the efficiency of screening [27,28]. Currently, the main mechanism of Nbs against pathogens is by blocking the binding of pathogens to host cells [29,30]. For example, researchers screened four Nbs against Shiga toxin-producing *Escherichia coli* (STEC) and enterotoxin-producing *E. coli* (ETEC) bacterial trichomonas protein F18, which competitively interfered with the binding of F18 to the blood group antigen receptor, thereby inhibiting the pathogen’s adherent infection in piglets [31]. A high-affinity Nb Abi-Se07 targeted virulence factor FliC of *Salmonella* was screened from an Nb phage library and was specific to adhere to the surface of *Salmonella* and inhibit its invasion into host cells [32]. In this study, we screened three specific Nbs from a naïve phage library using Sip proteins as targets. Among these, Nb30 demonstrated excellent Sip-binding activity in in vitro binding assays and was able to bind to GBS. To further validate the inhibitory activity, we evaluated the antibacterial activity of Nb30 by employing a GBS-infected tilapia model and quantified the copy number changes of GBS in spleen, liver and brain tissues of tilapia by qPCR. The results demonstrated that Nb30 significantly inhibited the proliferation of GBS in various tissues of tilapia. This inhibition may be attributed to the ability of Nb30 to bind to the Sip protein on GBS, thereby blocking the interaction between the Sip protein and the host receptor cells. Consequently, this hinders the adhesion of GBS to the host cells, which ultimately leads to the differences in survival rates of tilapia [33].

The TLR/MyD88/NF-κB signaling pathway is a key pathway in the inflammatory response system of fish, and it is widely present in a wide range of fish tissue cells and is involved in the pathogenesis and regulation of inflammatory and infectious diseases [34,35]. Toll-like receptors (TLRs) are a class of pattern-recognition receptors capable of triggering inflammatory immune responses in the body. All TLR proteins activate the MyD88-dependent pathway and generate a signaling cascade reaction with MyD88, during which the body will release a series of intermediate factors, such as irak4, traf6, etc., and ultimately activate the production of NF-κB family proteins involved in inflammatory response and cytotoxicity factors (Rela, NF-κB2, etc.) [36]. It has been shown that the expression of genes related to the TLR/MyD88/NF-κB signaling pathway is significantly up-regulated in tilapia after GBS infestation, leading to a severe inflammatory response in the fish [37,38,39]. Interestingly, the expression of genes related to the TLR/MyD88/NF-κB signaling pathway was down-regulated when Nb30 acted. In conclusion, Nb30 may attenuate the inflammatory response induced by GBS by inhibiting the TLR/MyD88/NF-κB signaling pathway.

It is well known that redox balance in the organism is essential for maintaining normal cellular activity and is closely related to a variety of physiopathological responses. However, when tilapia are infected with GBS, the attack of pathogenic bacteria induces cellular damage and a strong inflammatory response, leading to an imbalance between the oxidative and antioxidant systems caused by excessive production or insufficient removal of reactive oxygen species (ROS) [40]. Catalase (cat) and glutathione peroxidase (gpx) are important components of the antioxidant defense system in fish and play a key role in the balance between ROS production and antioxidants. When tilapia were infected with GBS, the gene expression levels of *cat* and *gpx* were significantly down-regulated [40]. Our study showed that Nb30 significantly improved the expression level of *cat*, *gpx* in tilapia, which is similar to the previous findings that specific antibodies could attenuate the oxidative damage caused by pathogenic bacterial invasion [41]. Lysozyme activity is an important marker of innate immunity in fish, and the innate immune response is characterized by its rapid response to the presence of pathogens, activation of cellular defense mechanisms and networks of action, including production of antimicrobial substances and proteins, and activation of nonclassical complement to help fish resist invasion by pathogens [42]. Bacteria often adhere to host cell receptors through surface structures, transforming into a state that allows them to form biofilms, thereby resisting antibody and complement penetration and evading phagocytosis by phagocytes. Therefore, developing drugs that target adhesins is a crucial measure in preventing and treating bacterial diseases [43]. Nb30 primarily targets the adhesin Sip of GBS. When Nb30 binds to GBS, it may interfere with GBS adhesion to tilapia tissues, causing GBS to become a free-floating state. This transformation facilitates the initiation of the host’s immune defense mechanisms, as free-floating bacteria lack physical barriers and are more easily phagocytosed by phagocytes such as neutrophils and macrophages [44,45]. Additionally, antibodies acting on the bacteria can activate the host’s complement system, enhancing immune clearance through opsonization [46]. Therefore, we speculate that Nb30 blocks GBS adhesion to host cell receptors, enhances the tilapia’s ability to capture free-floating bacteria, thereby promoting immune function and increased the expression of *c-type lysozyme* in GBS-infected tilapia, which helped tilapia to clear GBS from the body.

Cytochrome P450, a large superfamily of enzymes with heme as a cofactor, acts as a multifunctional biocatalyst and plays a key role in the detoxification of xenobiotics, the metabolism of exogenous and endogenous compounds, and homeostasis in vivo. In addition, several studies have shown that cytochrome P450 also plays an important role in immune defense. For instance, Zhang et al. identified a substantial number of cytochrome P450 genes from spotted catfish subjected to bacterial attack using RNA-seq, suggesting that these genes may play a significant role in defense against bacteria [47]. Reynaud et al. demonstrated that the expression of microsomal cytochrome P450 and related enzymes can be regulated when fish defense mechanisms are activated [48]. In the present study, Nb30 enhanced drug metabolism-cytochrome P450 and cytochrome P450 metabolic pathways to exogenous drugs in tilapia liver tissues, which may enhance the body’s immune response against GBS, thus exerting the biotransformation function and detoxification capacity of liver tissues.

A transcriptome analysis was conducted on the high-concentration treatment groups and the control groups, which yielded 637 differentially expressed genes (DEGs). Subsequent results revealed a series of DEGs implicated in substance metabolism and immune-related pathways, encompassing A scorbate and aldarate metabolism, Drug metabolism-cytochrome P450, Metabolism of xenobiotics by cytochrome P450 and chemokine signaling pathways. Ascorbate and aldarate metabolism represent a significant carbohydrate metabolic pathway in living organisms, with the capacity to protect cells from oxidative damage caused by aerobic metabolism and a range of pollutants. It has been found that ascorbate and aldarate metabolism were significantly inhibited when mud crabs were infected with mud crab diademovirus-1 (MCDV-1), which may facilitate the pathogen’s ability to infect the host [49]. In the present study, the expression of genes associated with ascorbic acid and aldaric acid metabolism was found to be significantly enhanced following the action of Nb30, a finding that aligns with previous studies that observed the up-regulation of antioxidant genes.

Chemokines are a class of signaling proteins or small cellular factors. In organisms, chemokines regulate leukocyte migration, influencing T cell secretion and are pivotal factors between innate and adaptive immunity [50]. A variety of CC-like and CXC-like factors, including CCL3, CCL19 & CXC14, were identified in our study, and there were different degrees of upward or downward trends of these chemokines, suggesting that the role of Nb30 for promoting immune responses in tilapia is complex. Interestingly, the present study revealed that GO enrichment analysis of immune response pathways identified multiple genes associated with antigen presentation and antimicrobial peptide expression, including mamu class II histocompatibility antigen, proteasome subunit beta type-9 (PSMB9), transmembrane protein 173 (TMEM173), and moronecidin. Notably, these genes exhibited significant downregulation in the control group compared to the treatment group. This phenomenon may be attributable to Nb30’s inhibitory effect on GBS adhesion to host cells, thereby enhancing the immune system’s capacity to capture free bacterial antigens. These findings are corroborated by the concomitant upregulation of c-type lysozyme expression, collectively supporting the critical role of Nb30 in activating both innate and adaptive immune responses in tilapia. In conclusion, transcriptomic analysis further demonstrated that Nb30 modulates multiple physiological processes in Nile tilapia, including antioxidant stress response, immune function, amino acid and protein synthesis, as well as energy metabolism. These comprehensive molecular changes collectively contribute to the improved survival and physiological homeostasis of fish under GBS challenge, highlighting Nbs’ multifaceted regulatory functions in enhancing fish disease resistance.

## 5. Conclusions

In this study, we focused on using the highly conserved adhesin Sip of GBS as a specific protein target, rather than the whole GBS bacterium, which may improve the antibacterial activity of nanobodies against GBS serum type variations. In a tilapia infection model, Nb30 effectively reduced GBS colonization in the liver, spleen, and brain of Nile tilapia, accompanied by a downregulation of TLR/MyD88/NF-κB pathway-related inflammatory genes and improved survival rates of infected fish. We also used transcriptomics to analyze the metabolic and immune functions of Nile tilapia, confirming that Nb30 alleviates oxidative stress, regulates immune responses, enhances amino acid/protein synthesis, and energy metabolism. In summary, this manuscript advances the study of nanobodies’ antibacterial mechanisms and provides a potential broad-spectrum protective nanobody against multiple GBS serotypes. Our findings offer a strategy for controlling GBS infection in tilapia and further validate the use of nanobodies as a sustainable antibiotic alternative for managing bacterial diseases in aquaculture. However, how to efficiently deliver Nbs into fish bodies is a problem that needs to be addressed in the future.

## Data Availability

The data supporting the findings of the study are available from the corresponding author upon reasonable request.

## Supplementary Materials

The following supporting information can be downloaded at: https://www.mdpi.com/article/10.3390/ani15213207/s1. Figure S1: Plasmid construction and protein expression of pET28a-Sip; Table S1: Sequences of primers for this study.
