# Supplementary material for: Protective Effect of Nanobodies Targeting Sip Protein Against Streptococcus agalactiae Infection in Tilapia (Oreochromis niloticus)
Source: Animals (Basel). 2025 Nov 4;15(21):3207. doi: 10.3390/ani15213207 (PMC12609131; doi:10.3390/ani15213207)
Supplement: Supplementary file 1 [file animals-15-03207-s001.zip › animals-3949850-supplementary.pdf]

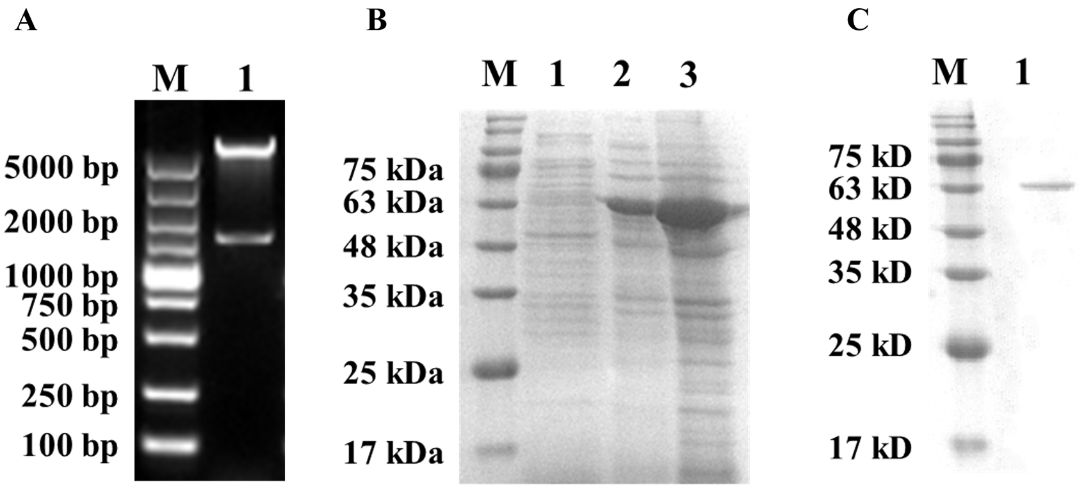

Figure S1. Plasmid construction and protein expression of pET28a-Sip. (A) Identification using restriction enzyme digestion; (B) SDS-PAGE analysis of the expression of Sip. M: protein marker, Lanes 1: empty pET-28a control, Lanes 2: supernatant, Lanes 3: precipitate; (C) SDS-PAGE analysis of the purified Sip.

Table S1. Sequences of primers for this study.

Primers	Primer sequences (from 5′ to 3′)
Onβ-Actin-R	CCACACAGTGCCCATCTACGA
Onβ-Actin-R	CCACGCTCTGTCAGGATCTTCA
Ontlr5-F	CGACATGGATACTGTTCATGG
Ontlr5-R	CTACAACGCCATCCAAACGC
Onmyd88-F	CAGGTTCCTGAGGTCGACAG
Onmyd8-R	CATTTCGTGGACGAACGCAA
OnIRAK4-F	CTCAATGACTGGGGGACCAC
OnIRAK4-R	TGGACTCGGGTAGCAGAACA
OnTRAF6-F	AAGAGCCACCTAGAAGAGCA
OnTRAF6-R	CTGACACTTCACACTGGCAA
OnRela-F	CAGATGAATACAGGCTGAGTGAGAA
OnRela-R	AGGTGCTGTCTATCTTGTGGAGTG
OnNF-κB2-F	GAACATCAGACCGACGACCA
OnNF-κB2-R	TCTCCGCCAGTTTCTTCCA
Onil-10-F	CTGCTAGATCAGTCCGTCGAA
Onil-10-R	GCAGAACCGTGTCCAGGTAA
Oncat-F	CCCAGCTCTTCATCCAGAAAC
Oncat-R	GCCTCCGCATTGTACTTCTT
Ongpx-F	ACCTTCATTCTCGCTACTCC
Ongpx-R	GCAGTTCTCCTGATGTCCAA
Onc-type lysozyme-F	CCCAGCTCTTCATCCAGAAAC
Onc-type lysozyme-R	GCCTCCGCATTGTACTTCTT
cfb-F	TAGCTTAGTTATCCCAAATCCC
cfb-R	TAAAGACTTCATTGCGTGCC
Sip-F	CGGGATCCATGAAAATGAATAAAAAGGTTACTATTGAC
Sip-R	ACGCGTCGACTTATTGTTAAAAATACGTGAACGTG
phiS3	CACAGGAAACAGCTATGACCATGATTA
psiR3	GCGTAACGATCTAAAGTTTTGTCG
Nbs-F	CCGGAATTCATGGCGGTGCAGCTGGTGGA
Nbs-R	CCCAAGCTTTCAGCGTGCGCCTGAGGAGACGG
